# An intrapancreatic accessory spleen presenting as a rapidly growing pancreatic mass after splenectomy in a patient with hereditary spherocytosis: a case report and literature review

**DOI:** 10.1093/jscr/rjy029

**Published:** 2018-02-23

**Authors:** Yukihiro Tatekawa

**Affiliations:** Department of Pediatric Surgery, Saku Central Hospital Advanced Care Center, 3400-28, Nakagomi, Saku-shi, Nagano 385-0051, Japan

## Abstract

The case of a 16-year-old boy with an intrapancreatic accessory spleen presenting as a rapidly growing pancreatic mass after splenectomy for splenomegaly due to hereditary spherocytosis is reported herein. When he was 15 years old, the patient visited at a hospital due to jaundice and radiological examinations showed a huge spleen with a 2-cm mass near or in the pancreatic tail. Sonazoid-enhanced ultrasonography showed hypervascularity in the mass located near the pancreatic tail, which was suspicious for an accessory spleen. During splenectomy by laparotomy, the mass could not be found by inspection or intraoperative ultrasonography. One year after the splenectomy, the mass grew rapidly to 4 cm. Laparoscopic surgery was performed to aid in the differential diagnosis of the mass, and a laparoscopic ultrasonogram revealed that the mass was located in the pancreatic tail. The patient underwent laparoscopic distal pancreatectomy and was discharged uneventfully on the 11th postoperative day.

## INTRODUCTION

An accessory spleen is found in 10–30% of patients at autopsy, and the splenic hilus is the most common site of an accessory spleen followed by the pancreatic tail [1–6]. In cases with an asymptomatic, well-delimited, tumor-like lesion in the pancreatic tail, the accessory spleen was smaller than 3 cm [1–3]. In cases of hematologic disease, laparoscopic exploration during splenectomy can miss 13–25% of accessory spleens and may lead to reoperation [1–6]. A case of an intrapancreatic accessory spleen (IPAS) presenting as a rapidly growing pancreatic mass after splenectomy in a patient with hereditary spherocytosis (HS) is reported herein.

## CASE REPORT

**Figure 1:**
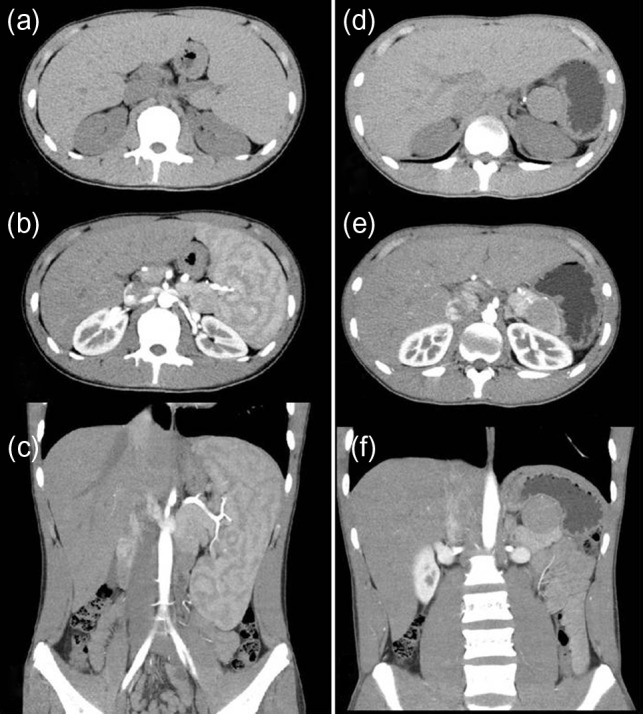
Computed tomography (CT) (before and after splenectomy). (**a**–**c**) Before splenectomy: Plain and enhanced CT shows a huge spleen and a 2-cm mass near or in the pancreatic tail. (**d**–**f**) After splenectomy: Plain and enhanced CT shows the mass in the pancreatic tail grows rapidly to 4 cm.

**Figure 2:**
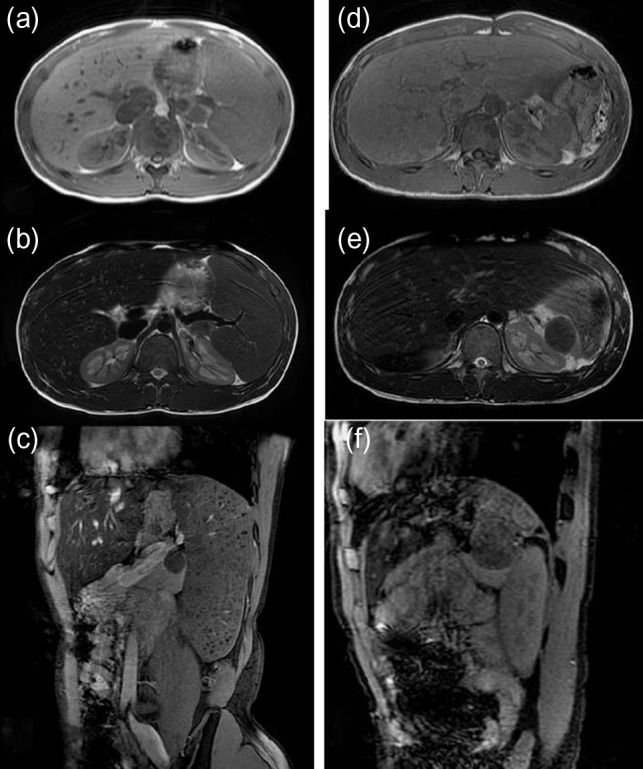
Magnetic resonance imaging (MRI) (before and after splenectomy). (**a**–**c**) Before splenectomy: MRI shows a huge spleen and a 2-cm mass in the pancreatic tail. (**d**–**f**) After splenectomy: MRI shows the mass in the pancreatic tail grows rapidly to 4 cm.

**Figure 3:**
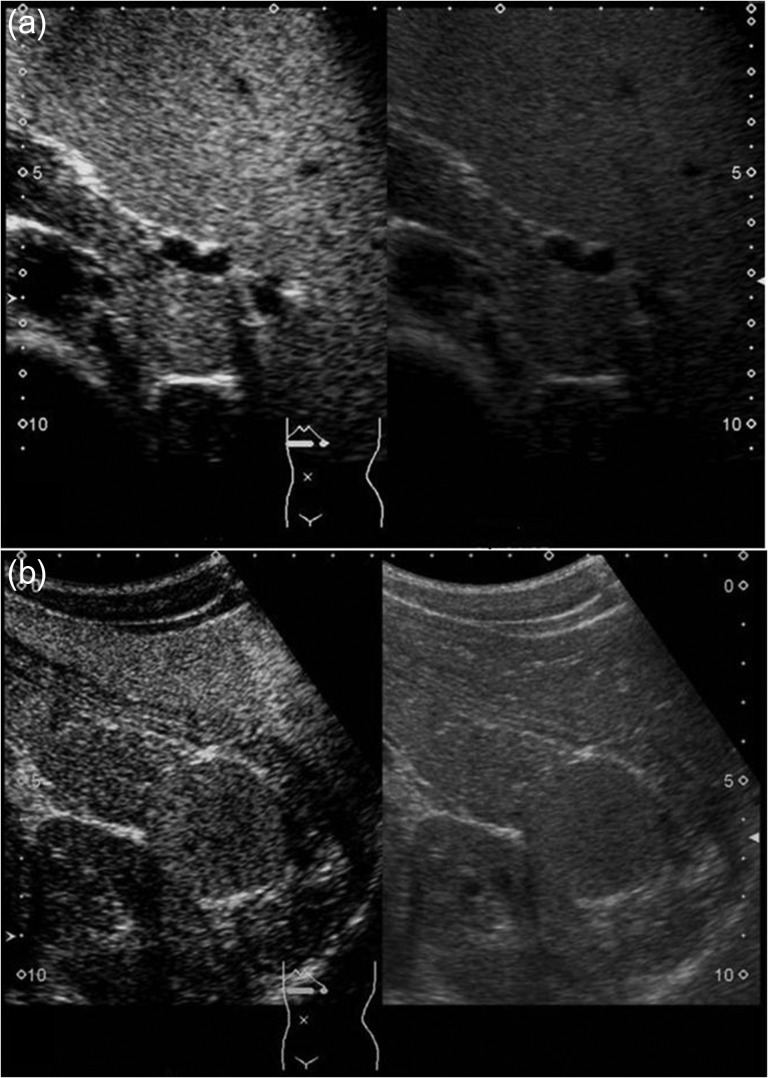
Sonazoid-enhanced ultrasonography Presplenectomy enhanced ultrasonography shows hypervascularity in the mass, located near the pancreatic tail and suspicious for an accessory spleen. The mass grows rapidly to 4 cm by 1 year after splenectomy (a: before splenectomy, b: after splenectomy).

**Figure 4:**
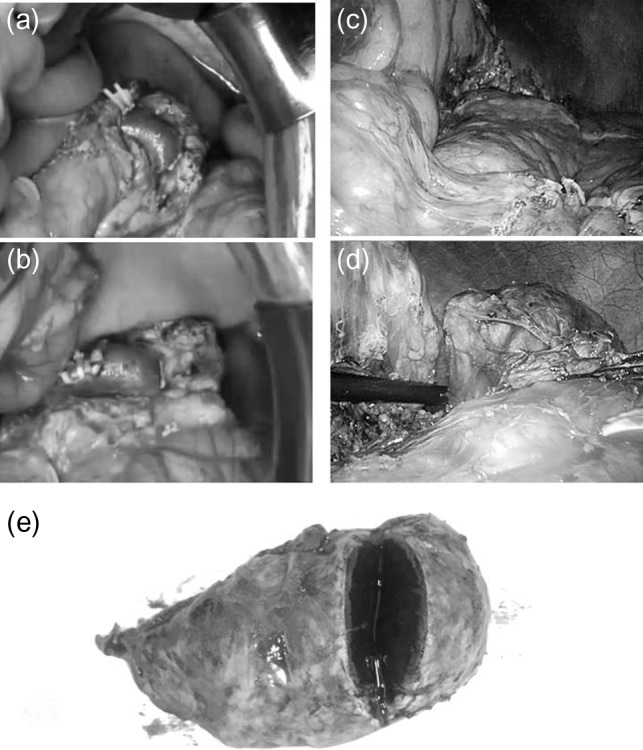
Intraoperative findings during splenectomy by laparotomy/laparoscopic distal pancreatectomy and the gross specimen The mass discovered during the preoperative examination is not found by inspection or intraoperative ultrasonography during splenectomy (**a**, **b**). But the laparoscopic ultrasonograhy reveals the mass in the pancreatic tail and a laparoscopic distal pancreatectomy is done (**c**, **d**). The gross specimen reveals a tumor, circumscribed by pancreatic tissue; histological evaluation shows an accessory spleen (**e**).

A 16-year-old boy was admitted with a rapidly growing pancreatic mass. He had received a diagnosis of HS based on the following: the need for a blood transfusion to treat anemia after birth, a history of hemolytic anemia, and a family history of HS. He had often undergone medical examinations for anemia and jaundice in junior and senior high school. When he was 15 years old, he visited our hospital because of the rapid onset of jaundice. Radiological examinations, such as computed tomography (CT) (Fig. 1a–c) and magnetic resonance imaging (MRI) (Fig. 2a–c), showed a huge spleen and a 2-cm mass near or in the pancreatic tail. Sonazoid-enhanced ultrasonography showed hypervascularity in the mass, which appeared to be located near the pancreatic tail and raised suspicions about an accessory spleen (Fig. 3a). The patient underwent a successful splenectomy by laparotomy, but the mass found on the preoperative examination could not be found by inspection and intraoperative ultrasonography (Fig. 4a and b). The mass grew rapidly to 4 cm one year after splenectomy, as shown on the radiological examinations (Figs 1d–f, 2d–f and 3b), but a recurrence of HS with anemia or jaundice did not develop. However, he underwent successful laparoscopic surgery to aid in the differential diagnosis. After the adhesions of the omentum to the abdominal wall were detached, the laparoscopic ultrasonogram revealed the mass in the pancreatic tail. He underwent laparoscopic distal pancreatectomy (Fig. 4c and d), and he had no clinical symptoms or signs of local infection or sepsis. A CT on the ninth postoperative day showed a small peripancreatic collection (Grade B: the clinical grading of postoperative pancreatic fistula). He was discharged on the 11th postoperative day. The resected specimen revealed the intrapancreatic mass that was covered with pancreatic tissues, and the intrapancreatic mass was an accessory spleen (Fig. 4e).

## DISCUSSION

Splenosis is usually found in the peritoneal cavity, retroperitoneum, thorax or distant sites, such as the brain. Isolated splenosis has been rarely reported in the liver, kidney, mesoappendix and stomach [7]. Pancreatic splenosis occurs usually after splenic trauma and surgery [8].

Because an IPAS does not usually require treatment, an accurate preoperative diagnosis to exclude a pancreatic neuroendocrine tumor (PNET), mucinous cystic neoplasm (MCN), solid pseudopapillary tumor or pancreatic metastatic tumor is important [1–3, 9]. For the radiological differential diagnosis, contrast-enhanced ultrasound has been used more frequently to diagnose spleen abnormalities. Levovist^®^ is an ultrasound contrast agent containing air microbubbles, which are trapped almost exclusively by the hepatic and splenic parenchyma in the delayed phase, also known as the ‘hepatosplenic phase’. In comparison with the pancreatic parenchyma, the IPAS appeared to be hyperechoic during all dynamic sonography phases. The echo enhancement of all IPAS, however, was identical to that of the spleen in all phases [1, 2]. Recently, Sonazoid^®^, a second-generation contrast agent, has been shown to produce more detailed contrast images than Levovist^®^ in patients with pancreatic diseases. In addition, detailed movements of microbubbles in the capillaries that were difficult to visualize with Levovist^®^ could be visualized repeatedly in real-time with Sonazoid^®^. Thus, improved diagnostic and differentiation capacities can be expected. It has been reported that Sonazoid-enhanced ultrasound might become a standard imaging technique for the diagnosis of an accessory spleen [10].

In the present case, the preoperative enhanced and plain CTs could not differentiate the location of the mass as being in the pancreas or near the pancreas, but an MRI revealed the intrapancreatic mass. On the other hand, contrast-enhanced ultrasound could detect the characteristic features of the mass. From these findings, it is clear that an MRI and contrast-enhanced ultrasound are useful to diagnose the IPAS.

A literature review revealed few reports about HS associated with an IPAS [4–6]. Additionally, an actual recurrence of HS induced by an accessory spleen has been rarely reported [4–6]. In the present case, a recurrence of HS with findings such as anemia or jaundice did not develop, but the IPAS had rapidly grown, necessitating resection.
